# pⅢ期非小细胞肺癌中miR-155的表达

**DOI:** 10.3779/j.issn.1009-3419.2014.05.10

**Published:** 2014-05-20

**Authors:** 轶 高, 圣灵 付, 文洋 江, 斌峰 李, 义涛 田, 向宁 付

**Affiliations:** 1 430030 武汉，华中科技大学同济医院胸外科 Department of Thoracic Surgery, Tongji Hospital, Huazhong University of Science and Technology, Wuhan 430030, China; 2 430060 武汉，武汉大学人民医院胸外科 Department of Thoracic Surgery, Renmin Hospital, Wuhan University, Wuhan 430060, China

**Keywords:** 肺肿瘤, miR-155, 预后, Lung neoplasms, MiR-155, Prognosis

## Abstract

**背景与目的:**

pⅢ期非小细胞肺癌（non-small cell lung cancer, NSCLC）患者5年生存率低于25%，需要寻找新的预后标志物，指导患者个体化治疗。MiR-155在许多肿瘤中表达上调，并对肿瘤有广泛的调控作用。本研究旨在探讨手术切除的pⅢ期NSCLC中miR-155的表达与预后的关系。

**方法:**

采用qRT-PCR法检测162例pⅢ期NSCLC手术患者福尔马林固定石蜡包埋组织（formalin-fixed, paraffin-embedded, FFPE）中miR-155的表达。

**结果:**

miR-155在pⅢ期NSCLC中表达与淋巴结转移程度正相关（*r*=0.169, *P*=0.032）。单因素生存分析显示在总体队列（*P* < 0.001）、鳞癌亚组（*P*=0.002）、腺癌亚组（*P*=0.003）中，miR-155高表达组的总体生存期（overall survival, OS）均低于miR-155低表达组。根据淋巴结转移程度分层分析显示在N0-1亚组中，miR-155高表达和低表达组间OS无统计学差异（*P*=0.319），在N2的患者中，miR-155高表达组OS显著低于miR-155低表达组（*P* < 0.001）。多因素生存分析显示miR-155高表达是影响患者预后的独立危险因素（RR=2.311, 95%CI: 1.479-3.611, *P* < 0.001）。

**结论:**

miR-15高表达不利于手术切除的pⅢ期NSCLC患者的总体生存，并且与淋巴结转移度正相关。MiR-155可作为评价pⅢ期NSCLC预后的生物标志物。

## Abstract (en)

**Background and objective:**

Despite undergoing curative resection, the 5-year survival rate for stage Ⅲ non-small cell lung cancer (NSCLC) patients is less than 25%. There is a need for biomarkers for prediction of survival and guiding individual therapy. MiR-155 is one of most commonly upregulated miRNAs in malignancies, and regulates multiple pro-oncogenic pathways. We aimed to investigate the prognostic impact of miR-155 in resected stage Ⅲ NSCLC patients.

**Methods:**

Tumor formalin-fixed, paraffin-embedded (FFPE) from 162 resected stage Ⅲ NSCLC patients were collected. Total RNA including miRNA was extracted, and qRT-PCR was used to determine the expression of miR-155.

**Results:**

Spearman rank correlation test showed a positive correlation between miR-155 expression and nodal status (*r*=0.169, *P*=0.032). MiR-155 expression had a significant prognostic impact in the total cohort (*P* < 0.001), in squamous cell carcinomas (*P*=0.002) and in adenocarcinomas (*P*=0.003). In N0-1 subgroup, miR-155 expression did not have a significant prognostic on overall survival in univariate analysis (*P*=0.319). In N2 subgroup, miR-155 had a negative prognostic effect on OS in univariate analysis (*P* < 0.001). *Cox* regression analysis revealed that miR-155 expression was unfavorable prognostic factors of OS (RR=2.311, 95%CI: 1.479-3.611, *P* < 0.001).

**Conclusion:**

High expression of miR-155 represents a valuable marker of poor clinical outcomes in patients with stage Ⅲ NSCLC.

肺癌是全球癌症相关死亡的首要原因^[1]^。非小细胞肺癌（non-small cell lung cancer, NSCLC）约占肺癌总数的80%。尽管肺癌已形成以手术治疗为主的综合治疗模式，但肺癌的5年生存率仍然不高，其中手术切除的pⅢ期NSCLC患者五年生存率只有9%-24%^[2]^。因此，迫切需要寻找新的肺癌生物标志物，指导肺癌的个体化治疗，提高患者的预后。MiRNA是一类长19 nt-25 nt的小非编码单链RNA，其通过对靶基因的转录后调控发挥生物学功能。MiRNA对肿瘤的发生、发展、转移各个环节都有调控作用^[3]^。例如，miR-21通过抑制PTEN的表达，促进非小细胞肺癌的生长、转移、放化疗耐药，其高表达不利于患者的预后^[4, 5]^。MiR-155已被报道在许多类型的肿瘤中表达上调，其对肿瘤细胞的生物学行为有广泛的调控作用。现有的研究^[6-9]^表明miR-155高表达不利于急性髓系白血病、乳腺癌、胰腺癌、肾透明细胞癌患者的预后，但其与NSCLC患者预后的关系仍不明确。本研究拟通过检测162例pⅢ期NSCLC福尔马林固定石蜡包埋组织（formalin-fixed, paraffin-embedded, FFPE）中miR-155的表达水平，并结合临床资料分析其与预后的关系，探讨其作为NSCLC预后标志物的价值。

## 1 资料与方法

### 1.1 临床资料和试剂

收集华中科技大学附属同济医院胸外科2006年10月-2012年9月手术切除、临床资料完整、尚有石蜡标本的pⅢ期原发NSCLC 162例。162例患者均经切片、HE染色，明确病理诊断（2003年WHO肺肿瘤组织学分类标准）。所有患者术前均未化疗或放疗。肿瘤分期根据2009年UICC肺癌TNM分期。患者年龄22岁-78岁，中位年龄55岁；男性133例，女性29例，pⅢa期131例，pⅢb期31例。随访时间为1个月-72个月，中位随访时间14.5个月。生存期的计算从手术日期起到随访日期或由于复发、转移而死亡的日期为止。miRNeasy FFPE Kit购自德国QIAGEN公司，RT-PCR试剂盒购自北京TransGen公司，SYBR green PCR master mix购于大连TAKARA公司，miR-155及U6逆转录和qRT-PCR引物购自广州锐博公司。

### 1.2 方法

#### 1.2.1 FFPE组织含miRNA总RNA的提取

每例蜡块标本切10 μm厚切片，去除接触空气的第1张切片，取4张切片用于总RNA提取。使用miRNeasy FFPE Kit石蜡包埋组织中的总RNA，按试剂说明书操作。使用Beckman Coulter DU700分光光度计测量OD_260_/OD_280_。

#### 1.2.2 qRT-PCR检测

取总RNA 2 μg进行cDNA第一链合成，根据逆转录试剂盒说明书合成模板。qRT-PCR采用SYBR green PCR mix体系，条件为95℃ 1 min变性，95℃ 15 s，60℃ 20 s，70℃ 15 s，40个循环，以U6为上样内参照，相对基因定量用*ΔC_T_*（*ΔC_TmiR-155_-C_TU6_*）方法计算。*ΔC_T_*值越大，表示miR-155表达量越低。将162例患者按miR-155相对表达量分为两组，*ΔC_T_*小于*ΔC_T_*平均值为miR-155高表达组，*ΔC_T_*大于*ΔC_T_*平均值为miR-155低表达组。

### 1.3 统计分析

SPSS 19.0软件包进行统计学处理分析。采用卡方检验进行高表达和低表达miR-155组间临床资料的差异分析；采用*Spearman*等级相关分析进行miR-155相对表达量和临床病理资料相关性分析；应用*Kaplan-Meier*法进行单因素生存分析，采用对数秩检验（*Log-rank*检验）；*Cox*比例风险回归模型进行多因素生存分析。*P* < 0.05为差异有统计学意义。

## 2 结果

### 2.1 石蜡包埋组织提取RNA及miR-155的检测

所有标本提取的RNA（包含miRNA）OD_260_/OD_280_值均在1.806-1.983之间。qRT-PCR反应的溶解曲线和扩增曲线见图 1A。

**1:**
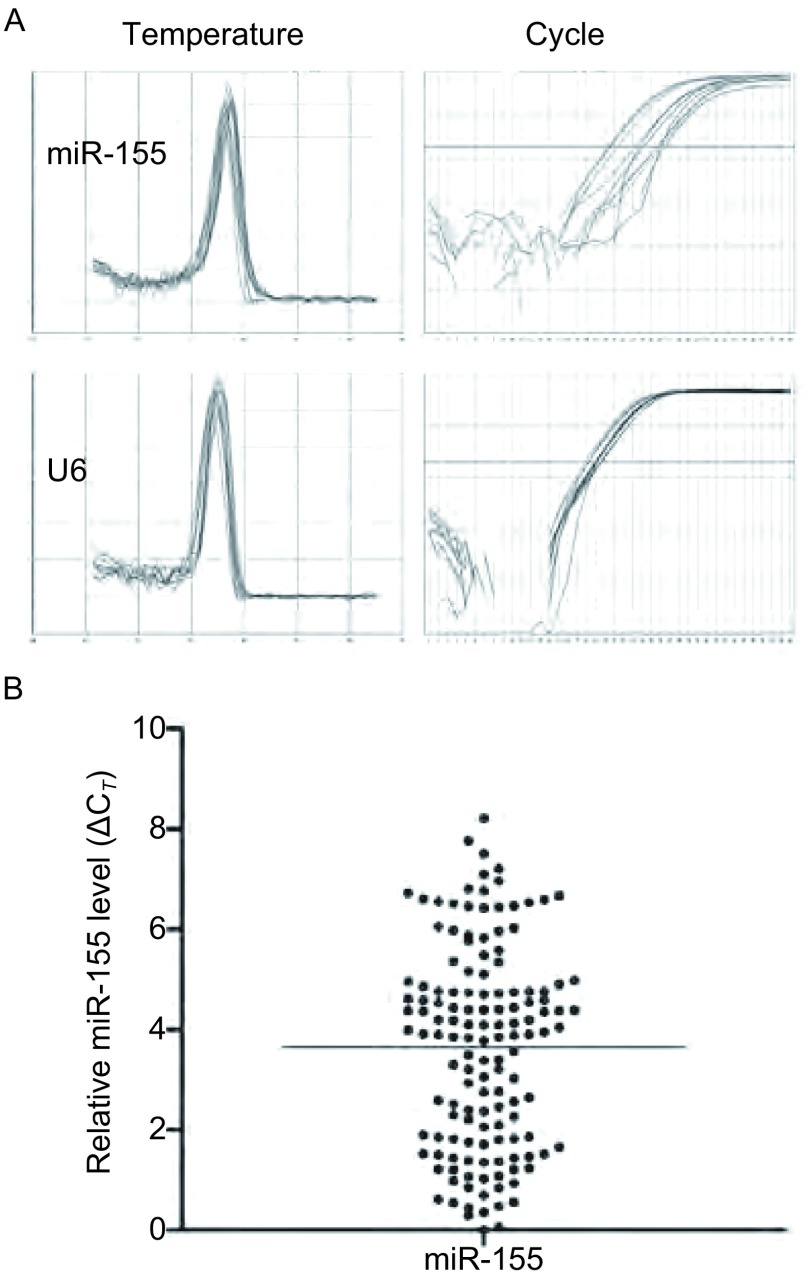
qRT-PCR检测miR-155和内参U6的表达 miR-155 and internal reference U6 were detected by qRT-PCR

### 2.2 miR-155在pⅢ期NSCLC中的表达

miR-155在162例pⅢ期NSCLC中均有表达。以U6为内参照，相对表达量*ΔC_T_*在0.01-8.21之间，平均值3.66±2.01（图 1B）。

### 2.3 miR-155组间临床资料差异分析

将162例患者按miR-155相对表达量分为两组，*ΔC_T_*小于3.66为miR-155高表达组（*n*=94），*ΔC_T_*大于3.66为miR-155低表达组（*n*=68）。统计学结果显示，淋巴结转移程度在miR-155高表达和低表达组间具有统计学差异（*P*=0.032），而年龄、性别、吸烟指数、肿瘤组织类型、TNM分期在两组之间的差异无统计学意义（表 1）。*Spearman*等级相关分析显示淋巴结转移程度与miR-155表达成正相关（*r*=0.169, *P*=0.032）（表 1）。

**1:** miR-155表达与手术切除的pⅢ期NSCLC的临床病理特征联系 Correlation of miR-155 expression with clinicopathologic characteristics of resected stage Ⅲ NSCLC

Characteristic	*n*	miR-155		*χ*^2^	*P*
		Low expression	High expression		
Age (year)				0.451	0.595
≤60	117	51	66		
>60	45	17	28		
Gender				0.237	0.682
Male	133	57	76		
Female	29	11	18		
Smoking index				0.223	0.744
≤400	63	25	38		
>400	99	43	56		
Histology				0.001	0.980
Squamous carcinoma	105	44	61		
Adenocarcinoma	57	24	33		
Pathological stage				1.486	0.312
Ⅲa	131	58	73		
Ⅲb	31	10	21		
T stage				1.738	0.619
1	2	0	2		
2	50	20	30		
3	46	21	25		
4	64	27	37		
Nodal status				^*^	0.032
0	28	18	10		
1	26	10	16		
2	108	40	68		
^*^: *Mann-Whitney U* test; NSCLC: non-small cell lung cancer.					

### 2.5 miR-155的表达与预后的关系

#### 2.5.1 *Kaplan-Meier*法单因素生存分析

对临床病理特征和miR-155的相对表达量进行*Kaplan-Meier*法单因素生存分析，结果显示：患者的性别（χ^2^=6.982, *P*=0.008）、病理组织类型(χ^2^=5.206, *P*=0.023）、TNM分期（χ^2^=10.730, *P*=0.001）、淋巴结转移程度（χ^2^=17.003, *P* < 0.001）、miR-155的表达（χ^2^=16.007, *P* < 0.001）是影响手术切除的pⅢ期NSCLC患者总体生存的重要预后因素，而年龄、PS评分、手术方式、术后是否进行放化疗与预后无关（表 2）。miR-155低表达组总体生存率高于高表达组，差异具有统计学意义（χ^2^=16.007, *P* < 0.001）（图 2）。我们根据组织类型、淋巴结状态、术后辅助治疗进行亚组分析（表 3）。在鳞癌亚组（χ^2^=9.634, *P*=0.002）和腺癌亚组（χ^2^=9.087, *P*=0.003）中，miR-155高表达组的总体生存率均低于miR-155低表达组。根据淋巴结状态将162例患者分成N0-1和N2两个亚组。统计学分析显示，在N0-1的亚组中，miR-155的表达对总体生存率无明显的影响（χ^2^=0.993, *P*=0.319）。而在N2亚组中，miR-155高表达组的总体生存率低于miR-155低表达组，差异具有统计学意义（χ^2^=13.773, *P* < 0.001）。在接受术后辅助治疗亚组（χ^2^=13.659, *P* < 0.001）和未接受术后辅助治疗亚组（χ^2^=4.499, *P*=0.034）中，miR-155高表达均不利于患者的预后。而且，低表达miR-155但未接受术后辅助治疗患者的总体生存率高于miR-155高表达但接受术后辅助治疗的患者（χ^2^=4.716, *P*=0.030）。

**2:** 不同预后因素对手术切除的pⅢ期NSCLC患者生存的影响 Influence of different prognostic factors on MST of resected stage Ⅲ NSCLC patients

Related factors	*n*	MST (month)	Range	*χ*^2^	*P*
Age (year)				0.138	0.710
≤60	116	24.0	0.9-72.0		
>60	45	28.0	1.0-62.0		
Gender				6.982	0.008
Male	133	25.0	1.0-72.0		
Female	29	13.0	0.9-61.0		
Performance status				0.906	0.341
0/1	154	24	0.9-72.0		
2	8	13	2.0-44.0		
Smoking index				1.247	0.264
≤400	63	20.0	0.9-72.0		
>400	99	24.0	1.0-62.0		
Histology				5.206	0.023
Squamous carcinoma	105	27.0	1.0-72.0		
Adenocarcinoma	57	19.0	0.9-61.0		
Surgical procedure				2.124	0.145
Sleeve resection	55	27.0	0.9-62.0		
Pneumonectomy	107	22.0	1.0-72.0		
Pathological stage				10.730	0.001
Ⅲa	131	25.0	0.9-72.0		
Ⅲb	31	11.0	2.0-44.0		
Nodal status				17.003	< 0.001
N0-1	54	48.0	1.0-62.0		
N2	108	19.0	0.9-72.0		
Adjuvant therapy				0.362	0.548
No	94	21.0	0.9-72.0		
Yes	68	27.0	1.0-65.0		
miR-155 expression				16.007	< 0.001
Low	68	29.0	1.0-72.0		
High	94	16.0	0.9-60.0		
MST: median survival time.					

**2:**
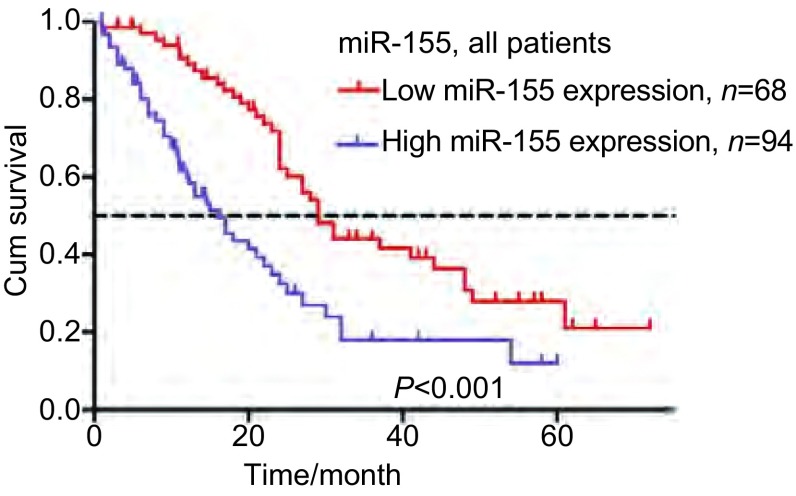
miR-155高表达组和低表达组的*Kaplan-Meier*累计生存时间曲线分析（*χ*^2^=16.007, *P* < 0.001） *Kaplan-Meier* survival curves according to miR-155 expression in total patients (*χ*^2^=16.007, *P* < 0.001)

**3:** miR-155的表达对预后影响的亚组分析 Prognostic impact of miR-155 expression in the histological and nodal status and adjuvant therapy subgroups

Characteristic	*n*	MST (month)	Range	*χ*^2^	*P*
SCC				9.634	0.002
Low	44	31	1.0-72.0		
High	61	20	1.0-60.0		
AC				9.087	0.003
Low	24	24	3.1-61.0		
High	33	10	0.9-58.0		
N0-1				0.993	0.319
Low	28	49	1.0-62.0		
High	26	32	1.3-60.0		
N2				13.773	P < 0.001
Low	40	24	3.0-72.0		
High	68	11	0.9-58.0		
Adjuvant therapy					
Yes	68			13.659	P < 0.001
Low	31	41	6.0-65.0		
High	37	17	1.0-54.0		
No	94			4.499	0.034
Low	37	27	1.0-72.0		
High	57	14	0.9-60.0		
SCC: squamous cell carcinomas; AC: adenocarcinomas.					

#### 2.5.2 *Cox*比例风险回归模型分析

*Kaplan-Meier*单因素分析发现，性别、病理组织类型、TNM分期、淋巴结状态、miR-155表达水平均与患者的总体生存率相关，故均纳入*Cox*多因素分析模型。采用条件向前法，结果显示：性别（RR=2.274, 95%CI: 1.353-3.823, *P*=0.002）、肿瘤T分期（RR=1.227, 95%CI: 1.033-1.458, *P*=0.020）、淋巴结状态（RR=3.552, 95%CI: 2.044-6.173, *P* < 0.001）、miR-155的表达水平（RR=2.311, 95%CI: 1.479-3.611, *P* < 0.001）是影响手术切除的Ⅲ期NSCLC患者预后的独立危险因素（表 4）。

**4:** 影响手术切除的pⅢ期NSCLC患者生存时间的*Cox*比例风险回归模型分析 Multivariate *Cox* proportional hazard analyses of prognostic factors for overall survival rates of resected stage Ⅲ NSCLC patients

Variables	Categories	B	SE	Wald	*P*	RR	95%CI	
							Lower	Upper
Gender	Female/Male	0.822	0.265	9.619	0.002	2.274	1.353	3.823
T stage		0.205	0.088	5.410	0.020	1.227	1.033	1.458
Nodal status	N2/N0-1	1.267	0.282	20.204	< 0.001	3.552	2.044	6.173
miR-155 expression	High/Low	0.838	0.228	13.536	< 0.001	2.311	1.479	3.611
RR: relative risk.								

## 3 讨论

TNM分期是目前评价肺癌术后生存的重要指标，但由于不同个体之间肿瘤的异质性，同一分期的不同患者预后仍不完全相同^[2]^。因此，需要寻找新的肿瘤生物标志物，指导个体化治疗，提高患者的预后。MiRNA因其广泛参与肿瘤起始和进展的调控引起了学者的关注。根据患者肿瘤组织和血液中miRNA的表达量作为肿瘤诊断和预后的标志物是可行和有效的^[3, 10]^。MiRNA检测最佳的标本材料是新鲜冰冻组织，但临床研究中新鲜冰冻组织难以大批量获取。石蜡包埋组织样本较新鲜冰冻组织容易获取和保存，但也存在mRNA降解的问题。miRNA单链长度只有19个-25个碱基，在标本固定过程中降解较少，并且较mRNA对化学和RNA酶诱导的降解稳定。已有研究^[11]^证明FFPE中miRNA的检测结果与新鲜冰冻组织相似。因此，FFPE是进行miRNA标本检测的理想材料^[12, 13]^。

MiR-155由人类21号染色体上的BIC基因编码，其表达受肿瘤抑制因子BRCA1负性调控。BRCA1通过与组蛋白去乙酰化酶2（histone deacetylase 2, HDAC2）形成复合物使miR-155启动子去乙酰化而抑制miR-155的表达^[14]^。研究^[15]^表明miR-155在许多肿瘤组织中高表达，而且有超过100个靶基因被证明受其调控，涉及JAK/STAT、TLR、ERK/MAPK、B细胞信号受体等多个关键信号通路。MiR-155异常高表达可以促进肿瘤细胞的生存、增殖、迁徙、侵袭、上皮间质转化、血管生成^[7, 15]^。MiR-15在肿瘤发生发展中的多重作用提示其可以作为肿瘤治疗靶点和预后因子。已有研究^[6-9]^证明miR-155表达升高是急性髓系白血病、乳腺癌、胰腺癌、肾透明细胞癌预后不良的危险因素。目前关于miR-155与NSCLC患者预后的关系尚存在争议。我们对162例pⅢ期NSCLC手术患者的研究显示，miR-155高表达对预后有不利影响，而且miR-155的高表达与淋巴结转移程度正相关。

Yanaihara等^[5]^使用液相杂交和qRT-PCR对104例NSCLC的miRNA表达谱进行检测，发现miR-155高表达与肺腺癌预后负相关。Raponi等^[16]^使用qRT-PCR对61例肺鳞癌手术患者标本进行检测，发现miR-155是肺鳞癌预后的不利因素。本研究通过qRT-PCR法检测162例pⅢ期非小细胞肺癌患者FFPE样本中miR-155的表达水平，发现miR-155高表达是pⅢ期NSCLC患者预后不良的独立危险因素。在肺鳞癌亚组和肺腺癌亚组中，miR-155与预后负相关，这与Yanaihara等^[5]^和Raponi等^[16]^研究基本一致。Donnem等^[17]^使用原位杂交检测335例Ⅰ期-Ⅲa期NSCLC miR-155的表达情况，发现miR-155高表达只在肺腺癌和淋巴结阴性肺鳞癌患者中显示出不利于预后的趋势，但差异未达到统计学意义，在肺鳞癌淋巴结阳性的患者反而对预后有利。我们的研究发现，miR-155与患者的淋巴结转移程度正相关，这与Chen等^[18]^在乳腺癌中的研究结果一致。淋巴结转移程度是影响患者预后的重要因素，本研究的结果显示不同miR-155表达水平组间淋巴结转移程度存在差异。因此，我们根据淋巴结转移程度进行分层分析。在N0和N1的pⅢ期NSCLC患者中，miR-155高表达对患者预后无明显影响。而在N2的患者中，miR-155高表达不利于患者的预后，差异有统计学意义。这提示miR-155可能在肺癌的迁徙转移过程中发挥重要作用，其表达的上调引起肿瘤转移能力增强，继而对患者预后产生不利影响。多因素生存分析显示miR-155高表达是影响pⅢ期NSCLC患者预后的独立危险因素。Donnem等^[17]^的研究中，肺鳞癌淋巴结阳性的患者总数仅有60例，样本数较少。本研究针对162例pⅢ期NSCLC手术患者，其中鳞癌患者105例，不同患者之间肿瘤分期差异更小。

我们还根据术后辅助治疗情况进行分层分析。在术后辅助治疗和未接受术后辅助治疗亚组中，miR-155高表达组的总体生存率均低于miR-155低表达组。而且，miR-155低表达的患者即使术后未行辅助治疗，其总体生存率仍优于接受术后辅助治疗的高表达miR-155的患者。这进一步提示了miR-155对肺癌患者预后的重要影响。本研究不足之处：术后辅助治疗资料不够详细，缺少疾病无进展生存期。

综上所述，miR-155高表达是手术切除的pⅢ期NSCLC患者预后的不利因素，miR-155高表达与淋巴结转移程度正相关。通过检测NSCLC患者手术切除标本中的miR-155的表达可以更好的判断肺癌的恶性程度和预后，指导肿瘤患者的个体化治疗。我们设想，通过靶向特定miRNA的寡核苷酸药物等技术抑制miR-155的表达^[19]^，抑制miR-155的生物学作用，改善肺癌患者的预后。
